# Design and Fuzzy Delphi Validation of an Augmented Reality Ergonomics Training Module for Manual Material Handling Workers

**DOI:** 10.21315/mjms-12-2025-902

**Published:** 2026-04-30

**Authors:** Fatimah Zahra Norman, Siti Munira Yasin, Mariam Mohamad, Meram Azzani

**Affiliations:** Department of Public Health Medicine, Faculty of Medicine, Universiti Teknologi MARA, Sungai Buloh Campus, Sungai Buloh, Selangor, Malaysia

**Keywords:** augmented reality, ergonomics, work-related musculoskeletal disorders, fuzzy Delphi, content validation

## Abstract

**Background:**

Manual material handling (MMH) workers are at high risk of developing work-related musculoskeletal disorders (WMSDs) resulting from repetitive lifting, awkward postures, and physically demanding tasks. Traditional ergonomics training methods are less engaging and may not effectively translate knowledge into safe workplace practices. This study aimed to develop and validate an Augmented Reality Physical Ergonomics (ARPE) training module to improve ergonomics knowledge, self-prevention behaviours, and self-evaluation skills among MMH workers.

**Methods:**

The ARPE module was structured around three domains (knowledge, self-prevention, and self-evaluation), comprising 25 subdomains identified through a systematic review of ergonomics guidelines and relevant studies. Content validation was performed using the fuzzy Delphi method with a panel of 10 multidisciplinary experts. The relevance and clarity of each item were evaluated by the experts on a 7-point Likert scale. Responses were converted into triangular fuzzy numbers and analysed using consensus criteria of threshold values (*d*) ≤ 0.20, ≥ 75% expert agreement and α-cut ≥ 0.50.

**Results:**

All 25 subdomains achieved expert consensus, confirming their relevance for ergonomics training among MMH workers. The validated framework emphasises knowledge of WMSDs and risk factors, proactive ergonomic practices, and workers’ ability to assess and adjust handling techniques.

**Conclusion:**

The content validity of the ARPE module was confirmed, and a comprehensive foundation for immersive ergonomics training was established. Pilot trials and long-term studies are needed to evaluate the module’s usability among MMH workers and to assess its practicality, user acceptance, and effectiveness in improving workplace ergonomic practices.

## Introduction

Manual material handling (MMH) tasks, such as lifting, lowering, pushing and carrying, are physically strenuous and associated with an increased risk of work-related musculoskeletal disorders (WMSDs). WMSDs are characterised by pain and movement restriction, resulting in decreased productivity and absenteeism. Globally, WMSDs are widespread: 60% to 92% of farmers experience at least one musculoskeletal condition (1), and healthcare professionals experience high rates of specific conditions, such as 21% for degenerative lumbar spine disease and 37% for hand osteoarthritis among dentists (2). In Malaysia, WMSDs account for approximately 25.5% of all compensation claims (3), and 94% of industrial packaging workers are at moderate risk of developing WMSDs (4). This burden is similar to that in other high-risk workplaces across countries and underscores the importance of implementing evidence-based ergonomic solutions, workplace policies and regulations to reduce WMSD risks and safeguard workers’ health.

Ergonomics programmes aim to align work demands with human capabilities to reduce injuries and enhance productivity (5). Traditional ergonomics training in Malaysia is typically delivered through lectures, printed materials or video demonstrations. However, such training has been widely criticised for its passive, one-directional delivery and limited capacity to promote behavioural change (6). Traditional approaches often lack contextualised demonstrations, interactive feedback and self-guided practice, resulting in poor knowledge retention and limited application of ergonomics principles to real-world tasks (7, 8). Workers frequently struggle to translate theoretical knowledge into safe handling practices, and the development of self-prevention and self-evaluation skills remains limited due to the lack of real-time corrective feedback (9). Thus, existing training approaches cannot always bridge the critical gap between ergonomics knowledge and the adoption of effective injury prevention behaviours (10, 11).

Advancements in Industry 4.0 technologies have offered new opportunities to update workplace ergonomics training. Technologies such as augmented reality (AR), virtual reality, wearable sensors and digital twins can help create immersive, interactive learning environments that enhance workers’ understanding of ergonomics principles. In particular, AR enables workers to visualise proper movements, practice safe handling, and receive instant feedback through realistic simulations (12). Compared with traditional training approaches, AR can enhance engagement, knowledge retention, task accuracy and postural awareness in sectors such as manufacturing, healthcare and aviation (9, 13). AR-based training promotes repeated practice and boosts learner motivation, both of which are crucial for cultivating safe manual handling habits (14). However, the adoption of these technologies in ergonomics training for MMH workers is limited, especially in Malaysia.

Developing an AR ergonomics module requires not only technological innovation but also rigorous content validation to ensure relevance, clarity and alignment with training objectives. The fuzzy Delphi method (FDM) offers a structured approach to achieving expert consensus by converting linguistic judgements into fuzzy numerical values, thereby reducing ambiguity in evaluation (15, 16). The FDM is increasingly used to validate educational and training modules, ensuring the development of systematic and reliable content.

Therefore, this study aimed to develop an Augmented Reality Physical Ergonomics (ARPE) training module for Malaysian MMH workers by integrating national guidelines and contemporary ergonomics evidence into an immersive mobile AR platform and validate its content. This module can enhance knowledge of ergonomics, promote preventive behaviours, and strengthen workers’ self-evaluation skills, with the aim of reducing WMSDs and improving workplace safety and productivity.

## Methods

### Study Design

This study employed the FDM to validate the content of an ARPE training module. The FDM was chosen to systematically gather, quantify and prioritise expert opinions on training content. By integrating fuzzy set theory with the classical Delphi technique, FDM converts linguistic responses into triangular fuzzy numbers (TFNs) and defuzzifies them to determine consensus (15, 16). This approach is well-suited for contexts involving expert judgement, uncertainty and subjectivity and has been widely applied in educational and module development research.

### Operationalisation of the Wheeler Curriculum Development Model

The ARPE training module was developed following the Wheeler Curriculum Development Model, which treats curriculum design as an ongoing cycle that involves setting aims and objectives, selecting learning experiences and content, organising them, and evaluating progress. This methodology was chosen to ensure pedagogical consistency and methodological accuracy in designing the ARPE module for MMH workers.

The first phase focused on establishing learning objectives to reduce the risk of WMSDs. These objectives aimed to improve knowledge of ergonomics, encourage preventive behaviours, and enhance workers’ ability to evaluate their manual handling techniques. They were grounded in Malaysian occupational safety standards and research on MMH risk factors and ergonomic solutions (18–20). The objectives fell into three main categories: knowledge, self-prevention and self-evaluation. The second and third phases focused on selecting suitable learning activities and training materials. AR was used to develop immersive, interactive experiences, including simulations, posture-visualisation overlays, and scenario-based demonstrations for correction. The training resources followed ergonomics standards and literature on WMSD prevention, highlighting biomechanical principles, hazard detection, safe lifting methods, and prevention strategies. The fourth stage focused on organising the selected content and learning experiences into structured AR modules that progressively developed ergonomics skills. Basic ergonomics principles were first introduced, followed by behavioural and reflective components, to support contextual learning and promote safe manual handling practices. In the final stage, the curriculum was evaluated using the FDM. Each item was reviewed for relevance, clarity and necessity by a multidisciplinary panel of experts. Items that met the predefined consensus criteria (*d* ≤ 0.20, expert agreement ≥ 75% and α-cut ≥ 0.50) (21–23) were retained, thereby ensuring that the ARPE module’s content was pedagogically robust and aligned with occupational health training standards.

### Item Generation from National Guidelines and Scientific Literature

A systematic review of relevant occupational safety guidelines and peer-reviewed scientific studies on MMH ergonomics and WMSD prevention was conducted to develop the initial set of items for the ARPE training module. This method ensured that the content was evidence-based, context-relevant and consistent with current occupational health and ergonomics intervention standards (24, 25).

A targeted literature review was undertaken using electronic databases such as Scopus, PubMed, Web of Science and Google Scholar to identify relevant sources, focusing on empirical studies, review articles and ergonomics intervention research published between 2020 and 2025. The following keywords related to occupational ergonomics and digital training were used to identify studies on ergonomics risk factors, training strategies and technology-supported learning methods aimed at reducing musculoskeletal injuries in workplaces (26, 27): “manual material handling,” “work-related musculoskeletal disorders,” “ergonomic intervention,” “ergonomic training,” “occupational ergonomics,” and “augmented reality training.” Furthermore, national and international occupational safety guidelines, including ergonomics risk assessment recommendations issued by the Department of Occupational Safety and Health Malaysia (28) and international occupational safety frameworks on manual handling and musculoskeletal injury prevention (26), were reviewed to ensure that the ARPE module reflected established workplace safety standards.

The retrieved literature and guidelines were subsequently screened for relevance to ergonomics training for MMH workers and WMSD prevention. Studies on ergonomics risk assessment, workplace biomechanics, safe manual handling techniques and technology-enhanced training interventions were prioritised, and those unrelated to occupational ergonomics or manual handling risks were excluded. The selected sources were analysed and synthesised to identify core ergonomics concepts, hazard identification strategies, preventive handling techniques and worker self-assessment practices relevant to industrial work settings (19). An initial pool of 25 potential content subdomains was generated, organised into the knowledge, self-prevention and self-evaluation pedagogical domains. The preliminary item pool served as the foundation for further refinement and expert validation using the FDM approach, which is widely applied in educational and health research to obtain structured expert consensus in the development of training modules and assessment instruments (29, 30).

### Item Refinement and Alignment with Learning Outcomes

A systematic refinement was conducted after the initial item pool was created to enhance clarity, relevance and alignment with the learning outcomes of the ARPE training module. The first draft was revised to remove duplicates, clarify wording and maintain conceptual consistency. Special attention was given to ensure that each item aligned with one of the three pedagogical domains. In this stage, unclear wording, overlapping concepts and overly technical terms were revised to improve clarity for MMH workers. The items were reviewed to confirm that they accurately represented the ergonomics skills targeted by the ARPE training module. This process ensured that the module’s learning objectives were aligned with the measurement items used in the subsequent evaluation. The refined items were reviewed by experts using the FDM. Each item was assessed for relevance, clarity and necessity in relation to ergonomics training and musculoskeletal injury prevention. The panel’s qualitative feedback was used to further refine item wording and confirm that each item accurately reflected the intended learning outcomes. This process enhanced the content validity and pedagogical coherence of the ARPE module before conducting the formal consensus analysis (30, 31).

### Expert Panel Selection and Calibration Interviews

Purposive sampling was used to select 10 subject-matter experts, aligning with the recommended panel size for homogeneous Delphi studies (32). Participants had at least 5 years of professional experience (33), expertise in ergonomics, occupational health, or instructional and training development, and a willingness to participate in at least 1 Delphi round. The panel included public health physicians, certified ergonomists, officers from the Department of Occupational Safety and Health, an industry safety and health officer and an instructional designer specialising in AR-based education technology.

Calibration interviews were conducted with three experts before the Delphi survey to assess the questionnaire’s clarity and relevance. Minor adjustments were made based on their feedback, including simplifying technical terms, refining item wording and improving alignment with the knowledge, self-prevention and self-evaluation domains. The revised questionnaire was shared with the entire expert panel for the fuzzy Delphi evaluation.

### Item Development and Content Domains

An initial pool of items was generated from national guidelines and empirical literature on MMH, ergonomics training and WMSD prevention (19, 20, 34–36). The items were organised into three pedagogical domains:

- Knowledge: Ergonomics principles, identification of WMSDs (signs, symptoms and complications) and recognition of high-risk MMH task characteristics.
- Self-prevention: Preventive behaviours, including microbreaks, ergonomic aids, safe lifting techniques, posture correction, adherence to procedures, and early self-reporting of symptoms.
- Self-evaluation: Assessment of personal technique and environment, identification of risk factors and application of corrective measures such as stretching or strengthening exercises.

The items were refined for clarity and alignment with learning outcomes before inclusion in the FDM questionnaire.

### Instrument Development and Data Collection

A structured FDM questionnaire was used to assess the relevance and necessity of each item and its suitability for delivery via an AR platform. Each item was rated by experts on a 7-point Likert scale, and qualitative feedback was obtained. Semistructured calibration interviews were conducted with a subset of experts to ensure clarity before distribution. The questionnaires were completed independently via email, and responses were converted into TFNs representing the minimum, most plausible and maximum values using a 7-point fuzzy linguistic scale (37).

### Delphi Procedure and Number of Rounds

Expert consensus was obtained using the FDM. Unlike the traditional Delphi method, which often requires multiple iterative rounds, the FDM reaches consensus in a single round by combining expert opinions with fuzzy set theory (38), thereby reducing the need for multiple rounds while maintaining methodological accuracy in measuring consensus. Only one round of expert evaluation was conducted in this study. The proposed ARPE module items were assessed for relevance, clarity and necessity by experts using a structured questionnaire on a 7-point Likert scale. Responses were converted into TFNs for analysis.

Consensus was determined using the FDM criteria: *d* ≤ 0.20, expert agreement ≥ 75% and an alpha-cut value ≥ 0.50. Items that met these standards were considered validated components of the ARPE training module. Given that the consensus thresholds were met in the initial round, no further Delphi cycles were needed. The use of a single-round FDM aligns with previous research that employed fuzzy Delphi techniques to develop educational and training modules (30).

### Delphi Data Collection Procedure

The Delphi questionnaire was administered to the expert panel via email after the calibration process and finalisation of the instrument. The experts were given two weeks to review and complete the questionnaire, ensuring sufficient time for careful evaluation of each proposed item. Reminder emails were sent after 1 week to encourage participation and ensure timely completion of the survey. All responses were collected within the specified timeframe and subsequently prepared for fuzzy analysis.

### Integration of Expert Qualitative Feedback

In addition to quantitative ratings, the experts provided qualitative comments to improve the clarity and relevance of the ARPE module items. This feedback was carefully reviewed by the research team and categorised into key themes, including terminology clarification, wording enhancements and alignment with ergonomics training goals. Ambiguous or conceptually overlapping items were revised to enhance clarity and appropriateness for MMH workers. These revised items were incorporated into the final item pool before the fuzzy consensus analysis. This approach improved the clarity and content validity of the ARPE training module (30).

### Data Analysis

Expert responses were analysed using the FDM to assess agreement on the proposed items for the ARPE training module. All fuzzy calculations were performed in Microsoft Excel (Microsoft Corporation, Redmond, WA, USA) using structured formulas for calculating TFNs and consensus metrics. Data analysis was performed as follows:

- Likert-to-fuzzy conversion: Ratings for each item were transformed into TFNs and averaged across experts (39).
- Consensus threshold (*d*) and expert agreement: The distance between each expert’s TFN and the group centroid was calculated (40). Items with *d* ≤ 0.20 and ≥ 75% expert agreement were retained (21).
- Defuzzification (α-cut): Items meeting the consensus threshold were defuzzified (23), and items with α-cut ≥ 0.50 were accepted (42).
- Item ranking: Accepted items were ranked based on defuzzified values to indicate their relative importance (43, 44).

This procedure ensured that only content with strong expert agreement was incorporated into the final ARPE module.

### Ethical Approval

This study was approved by the Research Ethics Committee of Universiti Teknologi MARA (Approval No. REC/11/2024 (PG/MR/550); date: 7 November 2024) and conducted in accordance with the Declaration of Helsinki, the International Council for Harmonisation Good Clinical Practice guidelines and Malaysian Good Clinical Practice standards. Participation of expert panel members was voluntary, and informed consent was obtained from all participants.

## Results

### Development and Validation of the ARPE Training Module

The ARPE module was structured around three domains—knowledge, self-prevention and self-evaluation—informed by ergonomics literature, expert input and observations of MMH workers performing tasks associated with WMSD risk. The knowledge domain covers fundamental ergonomics concepts, including body mechanics, WMSD identification and hazardous task recognition. The self-prevention domain emphasises proactive risk-reduction strategies, such as taking microbreaks, adjusting tools, practising optimal lifting techniques, maintaining proper posture and promptly reporting early WMSD symptoms. The self-evaluation domain enables workers to assess their own movements, identify ergonomics hazards and implement corrective practices, including stretching and strengthening exercises. All ARPE module items were embedded within interactive AR simulations and scenario-based learning components (Table 1).

### Content Validation Using the Fuzzy Delphi Method

The ARPE module’s content was validated using the FDM with a panel of 10 experts representing public health, ergonomics, occupational safety and health, industry practice, and instructional design. All items across the knowledge, self-prevention and self-evaluation domains were evaluated using a 7-point linguistic scale. The acceptance criteria included threshold values (*d*) ≤ 0.20, ≥ 75% expert agreement and defuzzified values (DV). All domains showed strong expert consensus:

- Knowledge domain: 8 items, *d* = 0.082, DV range = 0.86–0.93
- Self-prevention domain: 8 items, *d* = 0.074, expert agreement = 91%, DV range = 0.86–0.92
- Self-evaluation domain: 9 items, *d* = 0.080, DV range = 0.86–0.91

Given that all domains exceeded the acceptance thresholds, no items were removed, and all were included in the ARPE module. Furthermore, the proposed items met or surpassed the FDM acceptance criteria (Table 2). The knowledge domain exhibited the highest maximum DV (0.93), underscoring expert consensus on the fundamental importance of ergonomics understanding. The self-prevention and self-evaluation domains demonstrated strong agreement, supporting the need for behaviour-focused and reflective skill components in MMH ergonomics training. The FDM process confirmed the clarity, relevance and comprehensiveness of the ARPE module’s content. These validated items form the foundation for designing interactive AR training scenarios that are pedagogically sound, evidence-based and tailored to the ergonomic needs of MMH workers.

## Discussion

This study successfully developed and content-validated an ARPE training module for MMH workers in Malaysia. The module integrates foundational ergonomics knowledge, preventive practices and self-evaluation strategies to provide an evidence-based, immersive training tool. Expert consensus across the knowledge, self-prevention and self-evaluation domains indicates that the module’s structure is comprehensive, relevant and practically applicable. This module addresses the limited effectiveness of traditional manual handling training programmes in achieving sustained behavioural change and reducing WMSDs (45, 46).

Previous studies have consistently reported a gap between ergonomic knowledge and actual workplace practice. Although many ergonomics training programmes improve awareness and knowledge in the short term, their effects on sustained behavioural change and long-term reductions in WMSDs remain inconsistent and limited (24, 31, 45). This challenge has been widely discussed in systematic reviews examining training transfer and the effectiveness of workplace interventions. Several studies have reported that technique-focused ergonomics training, when implemented without adequate mechanical support or organisational intervention, may fail to produce sustained reductions in physical demand, behavioural risk, or injury rates (24, 45, 46). The ARPE module addresses this gap by embedding validated content within an interactive AR environment, supporting immersive, repeatable and hands-on learning experiences. The use of the FDM to validate the content of the ARPE module ensures scientific rigour and pedagogical coherence, addressing limitations identified in previous interventions that lacked structured, systematically validated curricula (47).

The high level of expert endorsement for the knowledge domain underscores the importance of establishing a cognitive foundation before skill acquisition. Understanding ergonomics principles, WMSD nature and biomechanical risk factors is crucial because deficits in these areas significantly contribute to unsafe handling practices and increased injury risk (48, 49). The ARPE module aligns with recommendations for comprehensive ergonomics education and increases the likelihood of safe behaviour adoption by combining theoretical knowledge with practical application.

The self-prevention domain strengthens the module by promoting proactive behaviours, such as correct lifting techniques, posture monitoring, microbreaks and early reporting of musculoskeletal symptoms. Integrated ergonomics and physical activity interventions are more effective in reducing musculoskeletal discomfort and improving flexibility than training alone. The emphasis on self-reporting addresses a common limitation in workplace safety programmes, where early musculoskeletal issues often go unreported until severe injury occurs (50).

The self-evaluation domain represents a significant advancement over traditional training programmes by enabling workers to monitor their own behaviours, recognise hazardous movements and implement corrective practices, such as stretching and strengthening exercises. Many conventional programmes omit self-regulation and reflective practice, thereby limiting their long-term impact (45). Embedding this domain within an immersive AR platform enables workers to engage in embodied learning, fostering greater self-awareness and promoting sustainable behaviour change.

Using AR technology in the module aligns with emerging evidence that immersive learning can improve knowledge retention, skill acquisition and engagement compared with conventional methods (46–49). Although research on AR in ergonomics remains limited, existing findings indicate that AR-based interventions can enhance hazard awareness and translate learning into safer work behaviours. Compared with sensor-based interventions, which are often expensive, intrusive and limited in real-world applicability (50), mobile AR offers a scalable, accessible alternative that integrates validated ergonomics content within a user-friendly platform.

By implementing an integrative approach, the ARPE module addresses cognitive, behavioural and self-regulatory dimensions of safe manual handling. This aligns with contemporary occupational health paradigms that advocate multimodal interventions combining knowledge, preventive behaviours, and environmental adaptation (41, 43). The ARPE module can help overcome key limitations of traditional training and technological interventions while enhancing worker engagement and long-term adherence to safe practices by providing an evidence-based, immersive and repeatable learning experience.

This study has some limitations. This was a content validation study. Therefore, it does not offer empirical evidence of behavioural changes, musculoskeletal outcomes or productivity gains among MMH workers. Pilot trials and long-term studies are needed to confirm the ARPE module’s success in practical settings. Moreover, the expert panel was mainly composed of Malaysian professionals, which could limit the applicability of the results to other cultural or regulatory environments. Finally, although AR offers immersive training, factors such as device availability, hardware variations, digital literacy levels, eye strain or fatigue and cognitive load may influence user experience and training outcomes. Future studies should thoroughly explore these issues to ensure effective implementation.

## Conclusion

This study developed and validated an ARPE training module for MMH workers, covering knowledge, self-prevention and self-evaluation. The FDM revealed strong agreement among experts on the clarity, relevance and completeness of all module parts. The ARPE module offers a validated, immersive and learner-focused ergonomics training framework that can help bridge the gap between knowledge and practice in manual handling education. The ability of this tool to reduce musculoskeletal risks and improve occupational health outcomes requires further testing through pilot programmes and effectiveness research in real workplaces.

## Figures and Tables

**Table 1:** Content of the ARPE training module

Domain	Subdomains
Knowledge	Ergonomic principles Definition of WMSDs (signs, symptoms, complications) Workplace and task factors that increase WMSD risk
Self-prevention	Proactive measures to prevent WMSDs Implementation of ergonomic principles and best practices (e.g., breaks, tool adjustment, proper lifting) Self-reporting of symptoms and consulting healthcare providers Work practice controls (safe work behaviour, compliance)
Self-evaluation	Proper manual handling techniques Identifying and addressing ergonomic risk factors Control initiatives (stretching, strengthening exercises)

**Table 2:** Fuzzy Delphi results for ARPE module content validation

Domain	No. of items	Threshold value (≤ 0.20)	Expert agreement (≥ 75%)	Defuzzified value range
Knowledge	8	0.082	≥ 75%	0.86–0.93
Self-prevention	8	0.074	91%	0.86–0.92
Self-evaluation	9	0.080	≥ 75%	0.86–0.91
